# Endoscopic Ultrasound-Guided Radiofrequency Ablation for Pancreatic Adenocarcinoma: A Scoping Review with Meta-Analysis

**DOI:** 10.3390/diagnostics15040437

**Published:** 2025-02-11

**Authors:** Cristian George Tieranu, Daniel Vasile Balaban, Daniela Tabacelia, Artsiom Klimko, Cristian Gheorghe, Stephen P. Pereira, Mariana Jinga, Adrian Saftoiu

**Affiliations:** 1 Faculty of Medicine, University of Medicine and Pharmacy Carol Davila, 020021 Bucharest, Romania; cristian.tieranu@umfcd.ro (C.G.T.); vasile.balaban@umfcd.ro (D.V.B.); daniela.tabacelia@drd.umfcd.ro (D.T.); cristian.gheorghe@umfcd.ro (C.G.); mariana.jinga@umfcd.ro (M.J.); 2 Elias Emergency University Hospital, 011461 Bucharest, Romania; 3 Central Military Emergency University Hospital, 010825 Bucharest, Romania; 4 Instituto Ecuatoriano de Enfermedades Digestivas (IECED), Guayaquil 090505, Ecuador; 5 Balgrist University Hospital, 8008 Zurich, Switzerland; artsiom.klimko@uzh.ch; 6 Fundeni Clinical Institute, 022328 Bucharest, Romania; 7 Institute for Liver & Digestive Health, University College London, London NW3 2PF, UK; stephen.pereira@ucl.ca.uk

**Keywords:** endoscopic ultrasound, radiofrequency ablation, pancreatic adenocarcinoma, technical success, adverse events

## Abstract

**Background:** Endoscopic ultrasound-guided radiofrequency ablation (EUS-RFA) has recently been proposed as an alternative treatment option for patients with unresectable pancreatic adenocarcinoma (uPDAC) or metastatic pancreatic adenocarcinoma (mPDAC). This review aims to evaluate the technical feasibility, safety, and clinical outcomes of EUS-RFA in treating PDAC, based on the available literature. **Methods:** Following the PRISMA-DTA guidelines, a comprehensive search of databases, including PubMed, Scopus, and the Cochrane Library, was conducted, focusing on studies reporting on EUS-RFA for PDAC. Articles involving human subjects diagnosed with PDAC and treated with EUS-RFA, written in English, and published up to 30 June 2024, were included. Key outcome measures such as technical success rate, adverse events, tumor response, and patient survival were extracted and analyzed. The review process involved title and abstract screening, followed by full-text review. A meta-analysis was performed for adverse event rates using a random-effects model. **Results:** We identified 11 studies according to our inclusion criteria, with a total of 137 patients with PDAC. Except for the initial experience with a lower technical success rate due to tumor-related stiffness, all subsequent studies reported a pooled success rate of 100%. Most studies referred to locally advanced or metastatic PDAC, while one reported EUS-RFA in resectable PDAC. A meta-analysis for adverse events was performed, indicating a pooled adverse event rate of 22.6% (95% confidence interval: 0.16–0.30), with the most common adverse event being mild abdominal pain. Severe complications were rare. One study reported a median progression-free survival (PFS) of 16.3 months. Overall survival and PFS were scarcely reported, with median overall survival ranging from 12 to 24 months, inferior to that of the standard approach for uPDAC consisting of neoadjuvant chemoradiotherapy followed by surgery. **Conclusions:** EUS-RFA is a technically feasible and safe procedure for treating uPDAC or mPDAC and is under investigation for use in resectable PDAC. Even though the short-term outcomes are encouraging, larger cohort studies are necessary to understand long-term efficacy and survival benefits, including progression-free survival.

## 1. Introduction

Pancreatic adenocarcinoma (PDAC) is one of the most lethal forms of cancer, defined by a poor prognosis, with limited treatment options and a lack of responsiveness to chemotherapy. Despite advances in surgical techniques and chemotherapy, the five-year survival rate for pancreatic cancer barely exceeds 10% [1]. The majority of patients are diagnosed at an advanced stage when the tumor is unresectable, underscoring the need to explore the field of alternative therapeutic approaches [2].

Recently, endoscopic ultrasound (EUS) has evolved from its role as a diagnostic tool into an important therapeutic alternative for pancreaticobiliary diseases, especially when endoscopic retrograde cholangiopancreatography (ERCP) fails [3]. Endoscopic ultrasound-guided radiofrequency ablation (EUS-RFA) has emerged as a promising minimally invasive therapy for use in pancreatic masses, after consistent reports of high efficacy and a good safety profile in neuroendocrine pancreatic tumors and pancreatic cystic lesions [4,5]. The initial experience with this technique in pig models showed promising results in terms of both safety and efficacy, allowing for precise targeting of the tumor while sparing surrounding healthy tissues [6,7,8,9,10]. In the early stages of using RFA for PDAC, most of the procedures were performed intraoperatively or percutaneously, with inconclusive results and significant morbidity [11,12,13,14,15,16]. The feasibility and safety profile of RFA for locally advanced pancreatic cancer using a surgical approach have been reported since 2010 [17,18]. These approaches often exhibited limitations due to local complications (biliary and pancreatic fistula, acute pancreatitis) and the technical difficulties of targeting tumors. Moreover, in a randomized controlled study, intraoperative RFA did not show any benefits in terms of overall survival (OS) and progression-free survival (PFS) when compared to conventional chemoradiotherapy in locally advanced PDAC [19]. However, the development of endoscopic ultrasound-guided RFA (EUS-RFA) has gradually shifted the perspective on this technique by providing real-time and precise targeting of pancreatic lesions, which enhanced the efficacy of tumor ablation while minimizing the damage to adjacent tissues and the risk of severe complications [20]. These findings have paved the way for EUS-RFA to become a more reliable tool in the management of PDAC.

The mechanisms through which EUS-RFA could potentially improve outcomes in pancreatic cancer patients are mostly related to tumor downstaging through coagulative necrosis and reduction in tumor mass [14]. Moreover, the disruption of tumor vasculature and the anti-angiogenic effect of thermal injury are also cited as mechanisms to control tumor microenvironments [12]. In addition to its contribution to reducing tumor burden via its cytoreductive effect, it appears that RFA also has an anti-cancer effect induced through immunomodulatory activity [21]. It is well recognized that the immunosuppressive nature and dense stroma of the PDAC tumor microenvironment contribute to its dismal prognosis by acting as a barrier to chemotherapeutic agents [22]. By disrupting the tumor microenvironment, local therapies have been theorized to potentially enhance the penetration of antitumoral treatments in PDAC [22,23,24]. Moreover, antigen exposure after RFA promotes immune activation and enhances immune response in tumoral lesions distant from the targeted site, known as the abscopal effect. In a murine model, Faraoni et al. showed that RFA can induce remodeling of the tumor microenvironment and increase antitumoral immunity, thus creating a window for the role of immunotherapy in PDAC [25].

This review aims to provide a comprehensive analysis of the current literature on EUS-RFA for pancreatic adenocarcinoma, focusing on key outcome measures such as technical success rate, adverse events, tumor response, and patient survival. By summarizing the findings from multiple studies, we seek to evaluate the overall effectiveness of EUS-RFA, identify methodological gaps, and propose areas for future research.

## 2. Materials and Methods

The research protocol was registered in the International Platform of Registered Systematic Review and Meta-Analysis Protocols (INPLASY) under the number 6973 (protocol ID: INPLASY2024100101; doi:10.37766/inplasy2024.10.0101). The literature search for the present study followed the Preferred Reporting Items for Systematic Review and Meta-Analysis of Diagnostic Test Accuracy Studies (PRISMA-DTA) guideline. A comprehensive search has been performed in medical databases such as PubMed Central, Scopus, and the Cochrane Library, focusing on articles reporting on the use of EUS-RFA in PDAC patients. The following MeSH (medical subject headings) terms and keywords were used: “Endoscopic Ultrasound”, “EUS”, “Endosonography”, “Radiofrequency Ablation”, “RFA”, “Radiofrequency Therapy”, “Pancreatic Neoplasms”, “Pancreatic Cancer”, and “Pancreatic Adenocarcinoma”. Specific search strings were constructed for each library to ensure thorough retrieval.

The inclusion criteria were as follows: (1) original research articles involving at least three human subjects (case series), written in English, and published (or available as “online first”) up until 30 June 2024; (2) studies involving patients diagnosed with pancreatic adenocarcinoma; (3) studies reporting the use of EUS-RFA; (4) studies providing relevant outcome measures such as technical success rate, adverse events, overall survival (OS), and progression-free survival (PFS). Exclusion criteria were related to the type of manuscript—letter to the editor, case reports, conference abstracts, manuscripts written in languages other than English, and studies on animal subjects. For the initial review stages (screening and duplicate exclusion), we employed Rayyan software (available online at www.rayyan.ai, accessed on 1 July 2024). After initial retrieval, duplicates were automatically identified and manually verified before exclusion, followed by the eligibility assessment of papers based on their relevance to the role of EUS-RFA therapy in PDAC. This phase involved a careful review of titles and abstracts by two team members (C.G.T. and D.V.B.), who voted independently for each article; voting conflicts were resolved by discussion between the two members, and consensus decision was made to include 10 articles. Selected articles were retrieved for full-text screening, and all eligible studies were then processed for data extraction. Furthermore, the references and citations list of the selected papers were manually searched for potential additional relevant studies which were not detected by the initial search. Pertinent studies that came to the attention of the authors through research platforms were also included, if eligible. This last source contributed an additional article, adding to the 10 previously selected, generating a total of 11 articles included in the final analysis.

**Figure 1:**
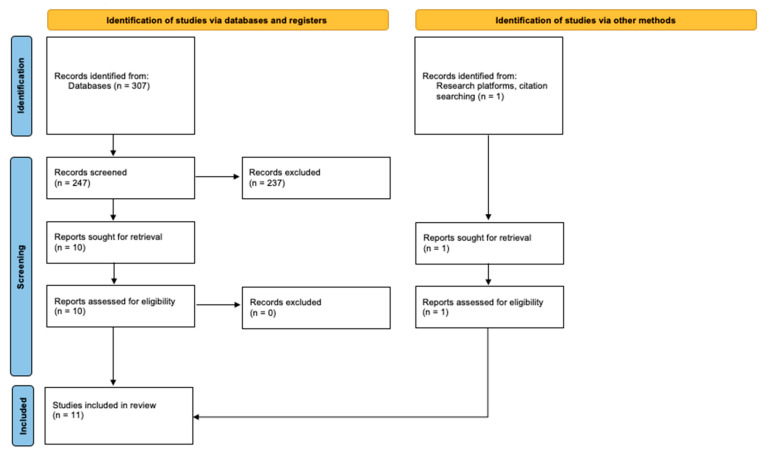
PRISMA flow diagram of the selection process.

The detailed process of study inclusion, based on the PRISMA methodology, is summarized in the following flow diagram (Figure 1).

### Statistical Analysis

The primary outcomes analyzed comprised the technical success rate and adverse events. Excepting the initial experience reported by Arcidiacono et al. [26], the technical success rate was consistently reported across all subsequent studies including PDAC lesions as 100%.

The secondary outcomes consisted of survival and tumor-related variables, such as progression-free survival (PFS), overall survival (OS), serum CA 19-9 level dynamics, and tumor size regression.

For the rates of adverse events, we calculated them as proportions of the total number of procedures in each study. A random-effects model was employed for the meta-analysis of adverse event rates due to the variability in reporting across studies, accounting for both within-study and between-study variances, to provide a more generalized estimate of adverse event rates. To handle cases with zero events, a continuity correction (adding 0.5 to each cell) was applied. The DerSimonian–Laird method (Tau-squared) was used to combine the odds ratios, accounting for between-study variability. Heterogeneity was assessed using Cochran’s Q test and quantified with the I^2^ statistic. A forest plot was generated to visualize individual study proportions and overall pooled estimates with corresponding confidence intervals. The meta-analysis was conducted using R software within the RStudio integrated development environment (Version 2024.9.0.375), chosen for its user-friendly interface and extensive facilities for data analysis and visualization [27,28].

## 3. Results

Based on the proposed inclusion criteria, 11 studies were selected for the final analysis. Data extracted from the studies included author names, publication year, number of patients, tumor location, tumor size and staging, type of RFA electrode used, power settings, number of RFA sessions, technical success rate, and adverse events. An overview of the studies included in the final analysis is presented in Table 1.

**Table 1:** Summary of studies reporting on EUS-RFA for PDAC.

Authors and Year	Study Type	No. of Patients	PDAC Staging	Tumor Location	Mean Tumor Size	Type of RFA Electrode	RF Generator	Power Settings	No. of RFA Sessions	Technical Success Rate	Survival	Adverse Events
Arcidiacono et al., 2012 [26]	P	22	Stage III LA-PDAC	Head and neck (16), uncinate process (2), body and tail (4)	35.7 mm (range 23–54 mm)	1.8 mm diameter 20 mm long cryotherm probe	VIO 300D RF-Surgery System ERBEKRYO CA system (Erbe Elektromedizin GmbH, Tübingen, Germany)	18 W	1 session	72.72%	Median 6 months in 13/16 pts	Abdominal pain—3 (18%) Minor duodenal bleeding—1 (6%) Rise in amylase level—3 (18%)
Song et al., 2015 [20]	P	6	LA-PDAC and mPDAC	Head (4), body (2)	3.8 cm (range 3 cm–9 cm)	18-gauge endoscopic RFA electrode	VIVA RF generator (STARmed, Koyang, Republic of Korea)	20 to 50 W	1–2 sessions	100%	Not evaluated	Mild abdominal pain in 2 patients, no major adverse events
Crinò et al., 2018 [29]	P	7	LA-PDAC	Head (2), uncinate process (2), body (3)	36 mm (range 22–67 mm)	18-gauge internally cooled electrode (EUSRA)	VIVA RF generator (STARmed, Seoul, Republic of Korea)	30 W	1–3 sessions	100%	Not reported	Mild abdominal pain in 3 patients, no major adverse events
Scopelliti et al., 2018 [30]	P	10	uPDAC non-metastatic	Head (4), body (6)	49.2 mm (range 35–75 mm)	18-gauge electrode (EUSRA)	VIVA RF generator (STARmed, Seoul, Republic of Korea)	20–30 W	1–2 passages	100%	Not evaluated	No major adverse events
Wang et al., 2021 [31]	R	11	uPDAC (7 LA-PDAC and 4 mPDAC)	Head (4), neck (3), body (3), tail (1)	27.9 mm (range 16.4–38 mm)	Habib EUS RFA catheter through 22G FNA needle	RITA System Generator 1500X (RITA Medical Systems, California, USA)	5–10 W	1–8 sessions	100%	1/11 pts survived at 12 months follow-up	Abdominal pain in 2 patients, no major adverse events
Oh D et al., 2022 [32]	P	22	uPDAC (14 LA-PDAC and 8 mPDAC)	Head (14), body (4), tail (3), resection margin (1)	38 mm (32.75–45 mm)	19-gauge RFA needle	VIVA RF generator (STARmed, Koyang, Republic of Korea)	50 W	5 sessions (median)	100%	Median OS 24.03 months	Early procedure-related adverse events—3.74% Abdominal pain—3 Peritonitis—1
Thosani et al., 2022 [33]	P	10	7 LA-PDAC, 3 mPDAC	Head (4), neck (2), body (2), tail (2)	39.2 mm (range 14—68 mm)	19- or 22-G through FNA probe, Habib 6500 ablation catheter (Boston Scientific, Marlborough, MA, USA)	Not specified	10–15 W	1–4 sessions	100%	Median survival 13.4 months	Mild abdominal pain in 12/22 treatments (55%), no major adverse events
Kongkam et al., 2023 [34]	P	14	stage III—1, IIIb—3, IV—10	Target lesions (30 sessions in 14 pts): head (5), body (11), neck (12), uncinate process (2)	59.7 ± 18.6 mm (IQR 39.8–66.1)	19-gauge RFA needle (EUSRA)	VIVA RF generator (STARmed, Koyang, Republic of Korea)	50 W	2.5 times per patient (1–4 times)	100%	6-month mortality rate 70%	Mild pancreatitis 1 (7.1%)
Napoléon et al., 2023 [35]	R	6 PDAC cases among 104 solid and cystic lesions	Not specified	Not reported specifically for the subgroup of PDAC pts	27 mm (13–60)	19-gauge RFA needle (EUSRA)	VIVA RF generator (STARmed, Koyang, Republic of Korea)	50 W	Not reported specifically for the subgroup of PDAC pts	97.1% for all lesions	Not reported specifically for the subgroup of PDAC pts	Overall AE 21.2% for all 100 lesions (NEN, metastasis, IPMN, SPEN)
Robles-Medranda et al., 2024 [36]	R	26	15 LA-PDAC, 11 mPDAC	Head (22), isthmus (2) body (1), tail (1)	39.5 mm (35.0–43.3)	19-gauge RFA needle	VIVA combo RF Generator System (TaeWoong Medical, Seoul, Republic of Korea)	50 W	1–3 sessions	100%	Median OS 7 (4–12) months	No major adverse events, mild pain in 3 patients
Wray et al., 2024 [37]	P	3	resectable PDAC	Head (3)	2.97 cm (2.7–3.2)	19-gauge RFA catheter	Not specified	Measured electrical impedance of 200 Ohm	2–3 sessions	100%	22.4 months	No adverse events observed related to the ablation

While the majority of studies focused exclusively on PDAC cases, Napoleon et al. investigated EUS-RFA in PDAC within a broader cohort that included both solid and cystic pancreatic neoplasms [35].

### 3.1. Patient Characteristics

The overall number of patients included in the selected studies was 137, with a variable number of RFA sessions, up to eight in the study by Wang et al. [31]. Most studies described 1–3 sessions per patient, whereas some authors performed several passages of the RFA needle in the same session to achieve adequate ablation, with heterogenous reporting, i.e., in Scopelliti et al., two needle passes were performed, with the same or different power-time settings, while in Kongkam et al., the EUS-RFA procedures were performed 2.5 times/patient, with 5.6 ± 2.9 needle passes/procedure [30,34].

Most frequently, the lesions targeted by EUS-RFA were located in the head of the pancreas, also encompassing the neck and the uncinate process (n = 103), followed by the body and tail of the pancreas (n = 43), and there was one case, in the study by Oh et al., of ablation in the resection margin after distal pancreatectomy [32]. Most of the studies included patients with unresectable PDAC, including locally-advanced and metastatic tumors, while Wray et al. reported EUS-RFA in three cases of resectable PDAC [37]. The median size of the lesions ablated was 38.17 mm, but RFA was even performed in large lesions up to 9 cm [20].

### 3.2. Technical Considerations

The initial studies used an 18-gauge RFA electrode, while the later research employed a 19-gauge RFA needle. The power settings (watts) varied between studies; some of the authors used 50 W, but others opted for lower ablation powers of 5–20 W. While the early work of Arcidiacono et al. showed a technical success rate of only 72.8% because of tumor and digestive wall stiffness [26], all subsequent studies including only PDAC lesions reported a success rate of 100%, even though there were studies including more than one session of EUS-RFA per patient. However, in the study by Crino et al. [29], RFA was not performed in one patient due to a large area of necrosis detected on contrast-enhanced imaging. Due to the uniformity of this outcome, a meta-analysis was considered futile.

### 3.3. Efficacy Analysis

Arcidiacono et al. [26] monitored tumor volume changes in patients undergoing EUS-RFA using CT scans. Of the 16 patients, only 6 displayed sufficient quality imaging to assess volume changes, and these patients were evaluated at two separate time points: once between 0 and 37 days post-ablation and again between 12 and 76 days. The results indicated a continuous reduction in tumor size over time. The median postoperative survival for 13 of the 16 patients who completed follow-up was 6 months. Another study, conducted by Oh et al. [32], analyzed overall survival (OS) and progression-free survival (PFS) in patients treated with EUS-RFA. The authors identified the time from tumor diagnosis to EUS-RFA and the tumor’s extent as negative impact factors on both OS and PFS. Additionally, they noted a marginally significant correlation between the number of EUS-RFA sessions and PFS (*p* = 0.051). The median OS and PFS in this study were superior to those previously reported in similar patient populations, suggesting that EUS-RFA may have a positive influence on these outcomes.

Furthermore, Wang et al. [31] proposed a different approach using EUS-RFA at a lower ablation power combined with multiple sessions. In their study, the efficacy outcomes included tumor size reduction, serum CA 19-9 levels, MRI diffusion-weighted imaging (DWI) apparent diffusion coefficient (ADC) values, and the percentage of the tumor area ablated one month after treatment. Two patients exhibited tumor size reduction, while five patients showed a decrease in serum CA 19-9 levels. One patient, who survived over 12 months, showed an increase in ADC values and a 20% ablated area, suggesting that EUS-RFA may contribute to prolonged survival. However, the overall survival rate in this cohort did not show a clear benefit from the procedure.

Similarly, Thosani et al. [33] performed EUS-RFA in 10 patients with unresectable pancreatic cancer, 7 with locally advanced pancreatic cancer (LAPC), and 3 with metastatic disease. The secondary endpoints included OS and serum CA 19-9 response. A total of 7 out of 10 patients with elevated pre-treatment CA 19-9 levels demonstrated significant reductions in serum CA 19-9 following the procedure. Follow-up imaging was available for nine patients, showing stable disease in two patients and tumor regression in seven, with a greater than 50% tumor size reduction in three cases. The median survival was 20.5 months, while post-RFA survival reached 13.4 months.

In the only randomized study, Kongkam et al. [34] explored the potential benefit of adding EUS-RFA to standard chemotherapy for advanced pancreatic cancer. Although no significant differences in survival were found between the groups, patients treated with EUS-RFA exhibited tumor necrosis in 100% of cases, according to imaging, and required lower doses of pain medication. Robles-Medranda et al. [36] also showed an improvement in oncological outcomes following EUS-RFA in advanced pancreatic cancer patients. They found a significant reduction in tumor size, with a greater than 50% diameter reduction in 5 of 11 patients. Additionally, the Eastern Cooperative Oncology Group (ECOG) score improved in all surviving patients, further suggesting that EUS-RFA may benefit patients with advanced disease.

Recently, Wray et al. [37] proposed a novel protocol for resectable PDAC, consisting of neoadjuvant chemotherapy and EUS-RFA, followed by surgery after a 4-week treatment break and then continuing with adjuvant chemotherapy. No anastomotic leak was observed after pancreaticoduodenectomy, all with R0 resections, in the three patients recruited. Also, there was no recurrence after a median of 13 months of follow-up.

### 3.4. Safety Profile

The safety of the procedure was remarkable, with no major adverse events being reported. Minor reactions, such as abdominal pain, were noted throughout several studies, along with one case of peritonitis [20,29,30,31,32,33,34,36]. The occurrence of minor early complications, such as abdominal pain or hyperamylasemia, led to a short prolongation of the hospital stay, while others, such as bleeding, required endoscopic hemostasis but without the need for blood transfusions [26]. Among the late complications reported, duodenal stricture requiring endoscopic stenting and auto-limited peripancreatic fluid collections was reported [26]. In the study by Kongham et al. [34], although no procedure-related major adverse events were reported, there were four withdrawals from the study due to disease progression (ascites, pulmonary embolism, and metastasis occurrence, along with deterioration in the ECOG score).

**Figure 2:**
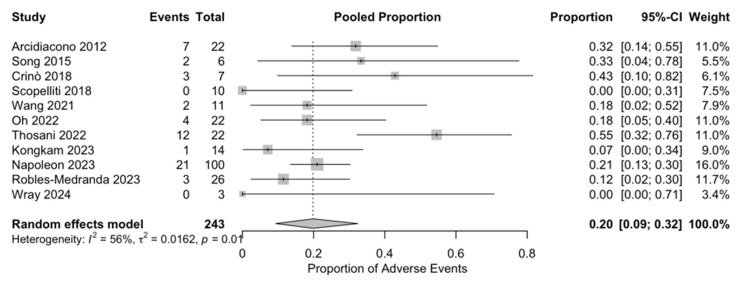
Random effects forest plot showing the pooled proportion of adverse events associated with EUS-RFA in patients with PDAC [20,26,29,30,31,32,33,34,35,36,37]. Each study’s proportion of adverse events is represented by a square, with the horizontal lines indicating the 95% confidence intervals. The size of each square reflects the weight of the study in the meta-analysis. The diamond at the bottom represents the overall pooled proportion and its 95% confidence interval.

Considering the variability in reporting adverse events, to better interpret the safety profile of the procedure, we performed a random-effects meta-analysis. The pooled estimate of adverse events from the random-effects model was calculated to be approximately 22.6% (95% confidence interval: 0.16–0.30). There was a moderate degree of heterogeneity in reporting adverse events among the studies (I^2^ = 52.4%, Q = 21.0, *p* = 0.012), mainly due to different definitions, patient populations, and study designs. Despite the observed variability, the results indicate that EUS-RFA was generally safe, with a low incidence of related adverse events (Figure 2).

### 3.5. Length of Hospital Stay

While some authors reported a predefined length of hospitalization according to the study protocol, such as five days in the paper by Arcidiacono et al. [26], others only noted a longer post-procedural observation in the recovery room, without the need for admission after EUS-RFA [33], and others did not quantify the length of hospital stay. In most studies, the average length of hospitalization was in the range of 3 days [29,30], with significant complications, such as mild pancreatitis, extending the hospital stay for 2 days [34].

## 4. Discussion

**Figure 3:**
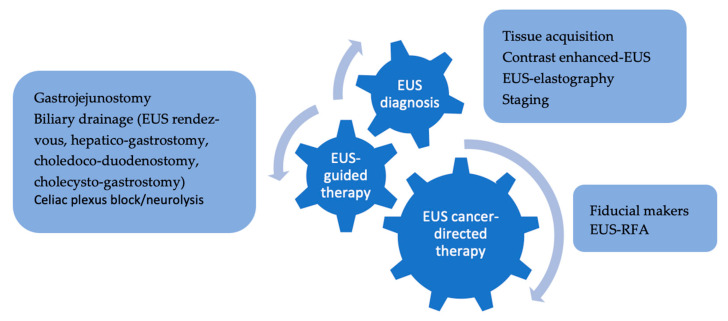
Role of EUS in the diagnosis and management of PDAC.

Initially developed as a diagnostic tool, EUS is currently being regarded as a one-stop shop for PDAC, providing tissue diagnosis, staging, drainage and derivative interventions (in case of biliary or gastric outlet obstruction), pain palliation (celiac plexus neurolysis), and also acting as platform for cancer-directed therapies, from fiducial placement for stereotactic body radiotherapy (SBRT) to the provision of ablative therapies—Figure 3 [38,39,40].

In broader clinical practice, EUS-RFA typically involves advancing a 19-gauge probe under real-time endoscopic ultrasound guidance into the target lesion. Ablation protocols vary but often use power settings of 5–50 W for durations ranging from 90 to 300 s per session. Care must be taken to monitor for tissue overheating and inadvertent injury to adjacent structures, especially major vessels or the pancreatic duct. Operator skill and familiarity with EUS-guided interventions are crucial for minimizing complications.

In this review, we assessed the technical feasibility, safety, and clinical outcomes of EUS-RFA for unresectable or metastatic pancreatic adenocarcinoma. The literature research per our protocol was restricted to 11 studies incorporating 137 patients. Our review encompassed studies with different designs and patient populations, from resectable PDAC to metastatic disease. Moreover, systemic therapies varied widely, with some patients receiving upfront chemotherapy, while others received none. RFA protocols were also diverse, mostly in terms of power settings and session counts, but initial studies with less than 100% technical success also employed a different probe. Such heterogeneity complicates pooled analyses and underscores the need for standardized protocols to better assess the efficacy and safety of EUS-RFA in this clinical scenario.

In the above-mentioned 11 selected studies, EUS-RFA was used either as a first treatment modality, concurrently, or after previous oncological treatments (FOLFIRINOX, gemcitabine, gemcitabine–capecitabine, gemcitabine–oxaliplatin, gemcitabine/Nab-Paclitaxel, gemcitabine/Abraxane-based chemotherapy, combination chemoradiotherapy, radiotherapy alone) [20,30,32,33,34,36]. The current treatment approach for LA-PDAC, consisting of induction chemotherapy (or chemoradiotherapy), with about one-third of patients proceeding to surgery, provides median OS rates in the range of 30 months [41,42].

Most studies have investigated the feasibility and safety of EUS-RFA in patients with pancreatic adenocarcinoma. Unfortunately, only part of the studies reported data on efficacy outcomes, including OS, PFS, and tumor size regression [26,31,32,33,34,36,37]. These studies reported high technical success rates and relatively low incidences of severe complications, underscoring the role of EUS-RFA as a promising option for patients with unresectable tumors [20,29,32]. Although not reported systematically in the included studies, oncological outcome measures such as OS provided in part of the studies are inferior to the current standard approach in LA-PDAC, consisting in neoadjuvant chemoradiotherapy followed by surgery, making EUS-RFA a palliative method in patients who are not surgical candidates [41].

Among the 11 studies included, only 4 [26,32,33,36] reported a clear median overall survival (OS) for patients with unresectable or metastatic PDAC. We excluded the cases from Wray et al., as these were patients with resectable PDAC. These studies encompassed a total of 74 RFA treated patients, with reported median OS values ranging from 6.0 to 24.03 months. The major heterogeneity in study design, patient population, and RFA protocols—combined with the lack of reported confidence intervals—precluded us from performing a formal meta-analysis employing standard statistical methods. However, we calculated a simple sample-size weighted average of the median OS across these four studies, with a pooled median OS of 12.7 months. There are several important limitations when interpreting this pooled median OS. First, it is based on data without matched controls, thus preventing direct comparison to other treatments. Second, patients varied in disease burden, from primarily locally advanced tumors in some studies to metastatic disease in others. Finally, none of the included studies reported a consistent set of covariates, such as performance status or prior chemotherapy, that could influence OS. Regarding PFS, only one study in our analysis reported a numerical median PFS [32] for PDAC patients undergoing EUS-RFA. Therefore, pooling data on progression-free survival was not possible. Future prospective trials should systematically report both OS and PFS with confidence intervals to allow for more rigorous meta-analyses.

The ability to employ real-time imaging during EUS-RFA, offering the opportunity to precisely target the tumor, most likely contributes to the high success rate of this method [6,7,26]. Moreover, applying RFA through EUS also provides the opportunity to implement other therapeutic measures, such as celiac plexus ablation, in the same session, as reported by Robles-Medranda et al. [36]. Different approaches in terms of power settings, RFA electrode types, and the number of RFA sessions did not show a significant influence on the technical feasibility of the treatment [29,31,32]. This consistent technical success rate throughout the studies underpins the feasibility of the procedure, even in the challenging clinical settings of locally advanced or metastatic PDAC [29,32,33].

Another important aspect that needs to be addressed with every new therapeutic option is safety. Similar to the technical success rate, the overall safety profile of EUS-RFA was consistently favorable, with most adverse events being mild (transient abdominal pain or hyperamylasemia). Differences in study definitions and reporting criteria likely contributed to heterogeneity (I^2^ = 52.4%). However, the pooled adverse event rate of 22.6% suggests that careful patient selection and periprocedural monitoring are essential for the timely diagnosis and management of these complications [20,33,34]. Severe complications, such as bleeding or duodenal strictures, were rare but should not be overlooked [26,29,32]. The relatively low incidence of major adverse events supports EUS-RFA as a relatively safe alternative for patients with limited options, even though long-term safety, particularly in combination with systemic therapies, remains to be further explored [32,34,36].

Although technical success and safety are crucial, the ultimate measure of a treatment modality is represented by clinical efficacy. Tumor response variables, such as reductions in tumor size and serum markers such as CA 19-9, showed encouraging results in several studies [30,31,32]. However, the limited availability of consistent data on overall survival (OS) and progression-free survival (PFS) highlights a major knowledge gap in the current literature. While one study reported a median PFS of 16.37 months, this outcome was not consistently evaluated across other trials [32]. The absence of standard outcome measures limits the ability to draw definitive conclusions about the long-term efficacy of EUS-RFA for PDAC patients compared to that of the current standard of care [20,29]. Regarding therapy monitoring using tumor biomarkers, it is important to note that up to 10% of the general population may have genetically undetectable CA 19-9 levels, potentially limiting its role as a reliable parameter [43]. Even among PDAC patients classified as non-secretors, a subset may still demonstrate abnormal CA 19-9 levels, adding further complexity [44]. It has been shown that CA 19-9 decrease during chemotherapy with gemcitabine predicts favorable survival outcomes in advanced pancreatic cancer [45]. However, this correlation of serum CA 19-9 dynamics before and after EUS-RFA with post-procedure survival was not demonstrated in the studies selected for this review. Thus, the serum decrease in CA 19-9 after EUS-RFA—while potentially encouraging—should be interpreted with caution and ideally, should be incorporated with imaging findings or other biomarkers in a broader analysis.

Other modalities focusing on locoregional therapy for PDAC, such as irreversible electroporation, did not show any benefits for OS compared to those of multimodal therapy, at the cost of significant post-interventional morbidity [46]. As with EUS-RFA, these local therapies remain non-curative and are intended for non-surgical candidates.

Tumor size before and after EUS-RFA was another efficacy outcome proposed by some authors. However, the lack of clear thresholds for defining morphological efficacy across the included studies made it unfeasible to elaborate any statistical comparisons. Only a subset of studies provided paired imaging data [26], but sample sizes were very small (n = 6 with evaluable imaging), and the results were described qualitatively as tumor volume reduction rather than with detailed numeric values. Thosani et al. [33] similarly noted that 7 of 10 patients exhibited measurable tumor regression, with 3 of them having a >50% tumor volume reduction, yet individual pre- and post-RFA paired dimensions were not reported. Robles-Medranda et al. [36] also found >50% tumor volume reduction in 5 out of 11 patients with advanced PDAC, again without individual lesion dimensions collected in a paired manner to serve as input data for statistical analyses. Overall, the limited data suggest a trend toward tumor size reduction in many patients, but the heterogeneity in outcome definitions limits a standard statistical approach at this time. Therefore, while there is evidence that EUS-RFA may induce local tumor shrinkage, a definitive pooled analysis or calculation of statistical significance (*p*-values) cannot be performed.

**Figure 4:**
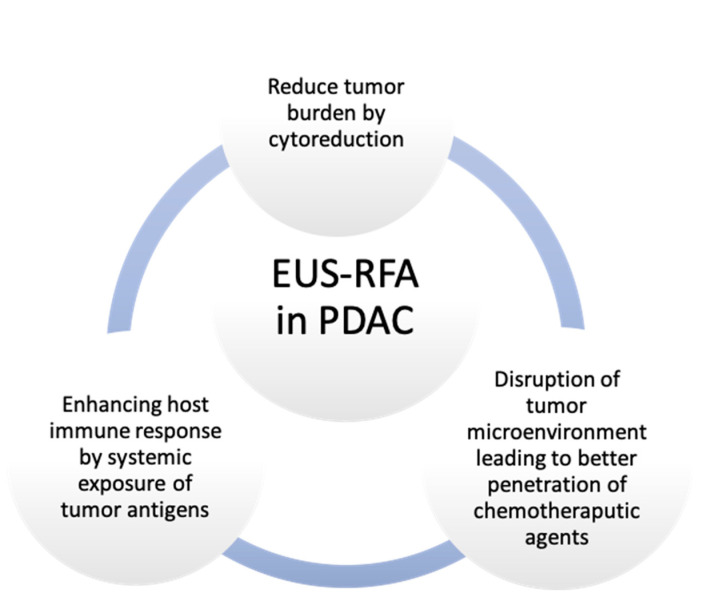
Potential benefits of EUS-RFA in PDAC therapy.

Besides the local control of the tumor through cytoreduction, the benefit of RFA comes from the immune modulation and the abscopal effect achieved by exposing tumor antigens [21,24,47,48,49]. Moreover, in the study by Song et al. [20], the authors revealed an increased blood flow around the RFA ablated area using contrast enhancement during EUS, which might create premises for better response to systemic chemotherapy. The potential benefits of EUS-RFA in PDAC treatment are summarized in Figure 4.

These findings generate intriguing questions regarding the possible role of combining EUS-RFA with immunotherapies, which could further contribute to better outcomes for patients with advanced PDAC [25,47]. Future research should concentrate on elucidating the mechanisms driving this immunomodulation and unraveling the synergistic potential of RFA added to novel systemic therapies [21,47,48].

Our review has several limitations. First, the studies included in this review were defined by small sample sizes and heterogeneous methodologies, which may affect the generalizability of the findings [32,33]. In addition, long-term data on survival outcomes after EUS-RFA remain limited [20,29]. A formal meta-analysis of overall survival (OS) and progression-free survival (PFS) was not feasible due to incomplete or heterogeneous survival data across the selected studies. Most studies lacked the necessary quantitative parameters, such as confidence intervals or event numbers. As more data become available from larger, prospective cohorts, a dedicated meta-analysis of OS and PFS would represent an important step in defining the long-term clinical utility of EUS-RFA. There is an urgent need for larger, well-designed prospective trials to provide more definitive evidence on the efficacy of EUS-RFA, particularly concerning long-term survival and its role in multimodal treatment strategies [32,33,49]. Furthermore, standardized definitions for the key outcome measures, such as tumor response, survival, and adverse events, are crucial to obtain more meaningful comparisons between studies [30,32,33].

In summary, while EUS-RFA appears to be a technically feasible and relatively safe technique for the treatment of PDAC, its integration into multimodal oncologic treatment protocols should be approached with caution, waiting for more conclusive evidence regarding its oncologic benefits. At present, EUS-RFA has proven to be a potential alternative in patients who are not surgical candidates, and further robust clinical trials are required to establish its efficacy. Future advancements in diagnostic technologies, including artificial intelligence-driven imaging, might contribute to refining patient selection for EUS-RFA and better delineating the treatment response to ablative therapy.

## 5. Conclusions

EUS-RFA appears to be a technically feasible and relatively safe procedure for patients with unresectable or metastatic PDAC. While short-term, procedure-related outcomes, such as high technical success rates and manageable adverse events, make EUS-RFA a potential therapeutic option, the lack of consistent long-term survival data underscores the need for further research. Future studies focusing on standardized reporting measures and exploring the integration of EUS-RFA with systemic therapies are essential to establish its clinical efficacy and to enhance the clinical outcomes for this aggressive malignancy.

## Data Availability

The data supporting this article are available within the manuscript.
